# Study of Secondhand Smoke Levels Pre and Post Implementation of the Comprehensive Smoking Ban in Mumbai

**DOI:** 10.4103/0970-0218.69269

**Published:** 2010-07

**Authors:** Aditi Deshpande, Priyanka Kudtarkar, Dhanashri Dhaware, Rohini Chowgule

**Affiliations:** Analytical and Industrial Hygiene Division, Indian Institute of Environmental Medicine, Mumbai, India

**Keywords:** Air quality, cigarette, hookah, secondhand smoke, smoking ban, tobacco, PM_2.5_ levels

## Abstract

**Objectives:**

This research was undertaken with the aim of assessing the indoor air quality in popular hospitality venues, as also to evaluate the effectiveness of the nationwide comprehensive public smoking ban. The analysis was split into two halves – baseline study taken up prior to implementation of the said ban on 2^nd^ October 2008, and the follow-up study after it came into effect.

**Materials and Methods:**

Twenty-five venues including five restaurants, fourteen resto-bars, two hookah (smoking water-pipe) cafes and four pubs were selected using a mix of random, convenience and purposeful sampling. Particulate matter (PM_2.5_) measurements at these venues were made using TSI SidePak AM510 Personal Aerosol Monitor.

**Results:**

The average PM_2.5_ level in venues where smoking was permitted prior to implementation of ban was found to be 669.95 *μ*g/m^3^ in the baseline study. Post ban, the average PM_2.5_ level in same test venues reduced to 240.8 *μ*g/m^3^. The hookah cafes were an exception as the average PM_2.5_ levels exceeded the permissible limits before as well as post ban.

**Conclusion:**

The baseline study showed that the hospitality venues had hazardous levels of PM_2.5_ particles arising from second-hand smoke prior to smoking ban. These decreased by a maximum of 64% after the law took effect. A substantial improvement in air quality at these venues post implementation of the smoking ban indicated the effectiveness of the law.

## Introduction

Second-hand smoke (SHS) from cigarettes is a known human toxin and carcinogen. SHS is a complex mixture of more than 4,000 compounds and is also referred to as involuntary smoking, tobacco smoke pollution, and passive smoking. Reports indicate that SHS causes lung cancer, heart disease and other health problems.(1,2) SHS mainly comprises side-stream cigarette smoke, the smoke released into the air from burning cigarettes between puffs.(3) Studies indicate that the fresh sidestream cigarette smoke is 3 to 4 times more toxic to laboratory animals than mainstream smoke i.e. the smoke inhaled by the smoker himself.(4)

SHS carcinogens include some polycyclic aromatic hydrocarbons (PAHs), heterocyclic aromatic amines, nitro compounds, organic compounds and inorganics like cadmium, chromium, hydrazine, nickel and polonium. Tobacco-specific nitrosamines (TSNAs), another group of highly carcinogenic compounds are formed exclusively from nicotine and other tobacco alkaloids. They have been linked to lung adenomas and adenocarcinomas, cancer of the nasal mucosa and liver.(5,6) TSNAs have been found in non-smokers exposed to second-hand smoke.(7)

Hookah smoke which is at par to smoke coming from 10 cigarettes also contributes to the SHS.(8) In mainstream cigarette smoke and mainstream hookah smoke plant-derived organic matter undergoes pyrolysis or volatilization, producing addictive nicotine as well as a number of the same toxicants from combustion. These include carbon monoxide (CO), tar, and myriad carcinogenic polycyclic aromatic hydrocarbons. All the above hazardous chemicals occur in the form of very fine suspended particles when released in significant amounts from burning cigarettes and are easily inhaled deep into the lungs. Suspended SHS consists of respirable suspended particles (RSPs) also known as PM_2.5_ (i.e. particulate matter equal to or less than 2.5 microns in diameter). Particles of large size result in less pulmonary absorption than smaller particles. Although larger particles may be inhaled into lungs, particles less than approximately 5 μm diameter are more likely to be inhaled and retained in the alveolus.(9)

A recent report of WHO asserts ‘the rule of 1000’ which states that a pollutant released indoor is one thousand times more likely to reach the lungs than a pollutant released outdoors. In recognition of the public health risk posed by second-hand tobacco smoke, the WHO has been encouraging countries to adopt smoke-free policies as a part of Framework Convention on Tobacco Control (FCTC). The Indian government has also been active in implementing various anti tobacco rules and laws like the Cigarette Act in 1975, Cigarette and Other Products Act in 2003, followed by the recent Act of Prohibition of Smoking in Public Places (2008).(10) So, in this study we aimed at the effectiveness of this law by assessing tobacco smoke derived fine particulate matter before and after implementation of the comprehensive smoking ban at hospitality venues. Mumbai city was chosen as the target because of its large pool of successful entrepreneurs and large number of popular hospitality spots.

## Materials and Methods

### Sampling period

Indoor air quality was assessed in 25 hospitality venues in Mumbai before and after implementation of the comprehensive public smoking ban in India on October 2, 2008. The pre ban measurement of fine particulates (PM_2.5_) was conducted from September 22 to October 1, 2008 and post ban from December 26 to January 11, 2009 at same venues in Mumbai.

### Selection of venues

**Figure 1:**
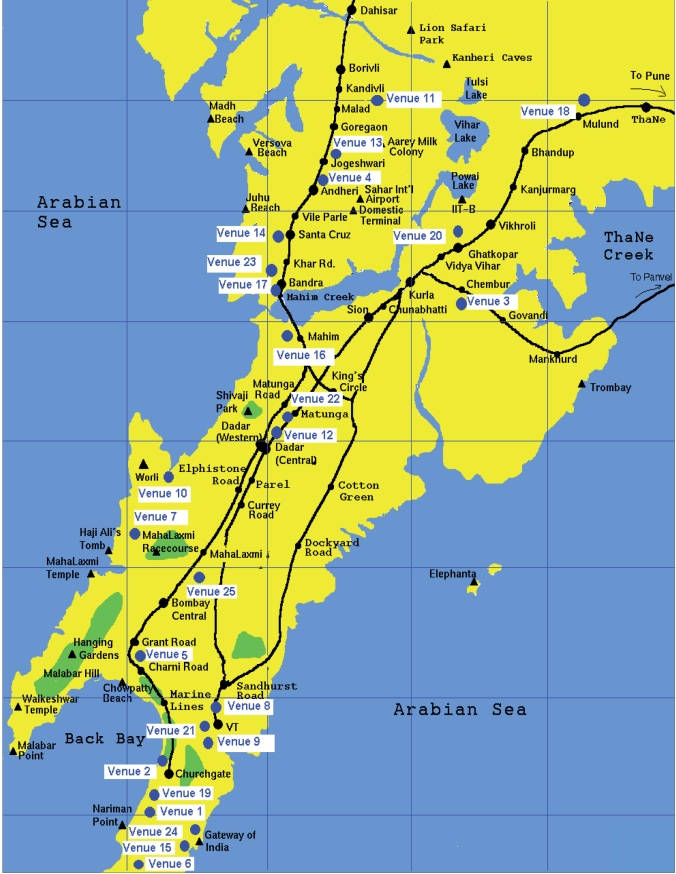
Mumbai city map showing study venue locations (●=Venue Number)

A comprehensive list of venues was created, which straddled both socio-economic and service variety ranges. From this list, 25 locations were selected that included 5 restaurants as control sites and remaining 20 sites included 14 bars, 2 hookah parlors and 4 pubs. The venues were selected on the basis of geographical location so as to cover maximum city, type of restaurants such as Udipi, Irani, 3-star, 5-star. 20 test venues were selected such that a representative of all grades of restaurants from each suburb in Mumbai could be included. The geographical expanse from the separation between these sites is depicted in the Mumbai city map in Figure 1. Venues where smoking was prohibited (6, 7, 9, 21, 22) were chosen for control readings.

### Sampling time

The sampling was performed for a minimum of 60 minutes each, at lunch time, during happy hours and dinner time, when the venues were crowded, that included restaurants, bars, cafes and pubs.

### Sample collection

A TSI SidePak AM510 Personal Aerosol Monitor (TSI, Inc., St. Paul, MN) was used to sample and record the levels of particulate matter in the air as in earlier studies.(11) Sampling was carried out in a tactful manner so that the occupants’ normal behavior would not be hampered. The monitor was often located in a central location on a table or bar so the air being sampled was within normal breathing zone. For each venue, the first and last minute of logged data were removed because they are averaged with outdoors and entryway air. The remaining data points were averaged using the TrakPro software to provide an average PM_2.5_ concentration within the venue. Additionally, the number of people smoking and volume of the room were noted to check the smoker density.

## Results

Indoor air quality was assessed in 25 hospitality venues across Mumbai city. In the baseline study, fine particulates (PM_2.5_) were measured through late September 2008 prior to the implementation of comprehensive smoking ban in India. The average PM_2.5_ level across the 25 locations was compared to the National Ambient Air Quality Standard (NAAQS) of 35 μg/m^3^. The mean concentration at five control locations was found to be 47 μg/m^3^, which was not far greater than the NAAQS limit. However, at the remaining test locations, the average was 669.95 μg/m^3^, almost 20 times higher.

The same 25 venues were reassessed in December 2008 as part of the follow-up study post-implementation of the smoking ban, which was enforced with effect from 2^nd^ October 2008. The average PM_2.5_ level at the venues had reduced to 240.8 μg/m^3^ from 669.95 μg/m^3^ recorded in the baseline study – a 64% decrease.

**Figure 2:**
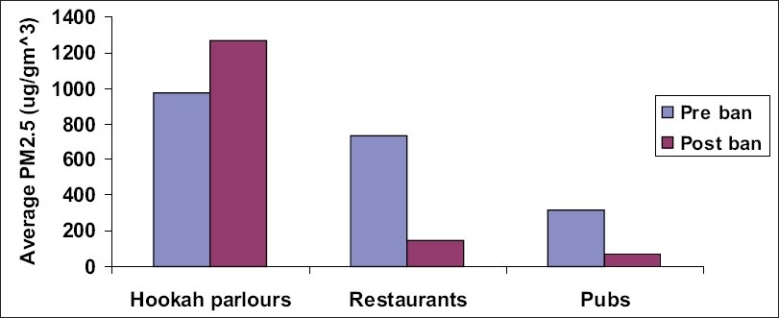
Summary of average PM_2.5_ levels

The results showed that levels of PM_2.5_ measured after the ban were lower than those measured before the implementation of smoking ban. Nevertheless, the concentration recorded post-smoking ban was 240.8 μg/m^3^, which is still almost 7 times the NAAQS. Notable exceptions were hookah parlors where the PM_2.5_ concentrations increased. This is possibly due to an exodus of smokers from their customary venues to hookah parlors, since these parlors were clearly violating the law under the excuse that the flavored hookah’s being served did not contain any nicotine. The Hookah parlors also fall under the category of smoke-free places as per the Prohibition of Smoking Act for public places. Comparison of PM_2.5_ concentration before and after the ban in all three hospitality categories is graphically plotted in Figure 2. Recent studies have reported that the air quality has indeed improved in bars, pubs and restaurants with smoke-free laws.(12)

**Figure 3:**
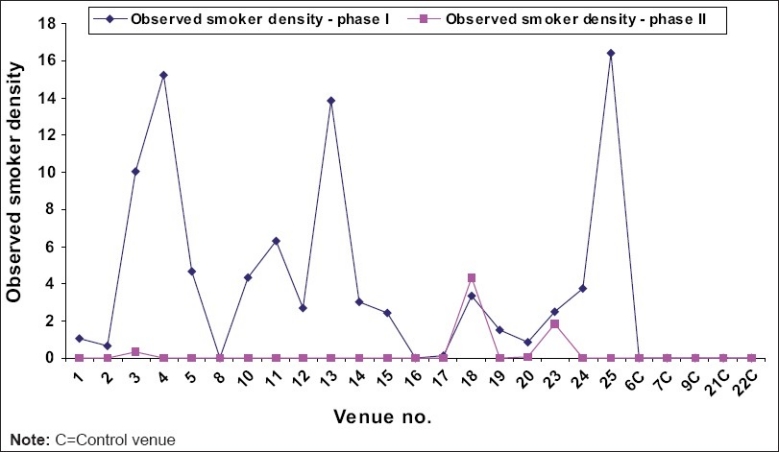
Observed smoker density pre and post-smoking ban

Active smoker density averaged across all venues in the follow-up study was 1.6 as compared to baseline 4.8. Prior to prohibition, smokers were noticed in pockets at almost all locations. But after the ban, no smokers were seen at any venues except the two hookah parlors. Smoker density of two hookah parlors after ban implementation was observed as 3.08 burning cigarettes per 100 cubic meters volume. Smoker densities before and after the ban are compared in Figure 3.

Among restaurants, control venue 7 showed PM_2.5_ concentration as high as 182 μg/m^3^ in the follow-up study and 128 μg/m^3^ baseline. This was attributable to an open kitchen serving orthodox continental and Indian tastes. The highest PM_2.5_ concentration 435 μg/ m^3^ was recorded at Test Venue 4 (a restaurant), although no smokers were sighted. This too was attributable to several factors like an open tandoor kitchen, its location amidst high traffic density, relatively low air volume, as also its being particularly crowded. Venue 5 also showed a remarkably high concentration (402 μg/m^3^), and the reasons are similar. Here too, no smokers were noticed.

**Figure 4:**
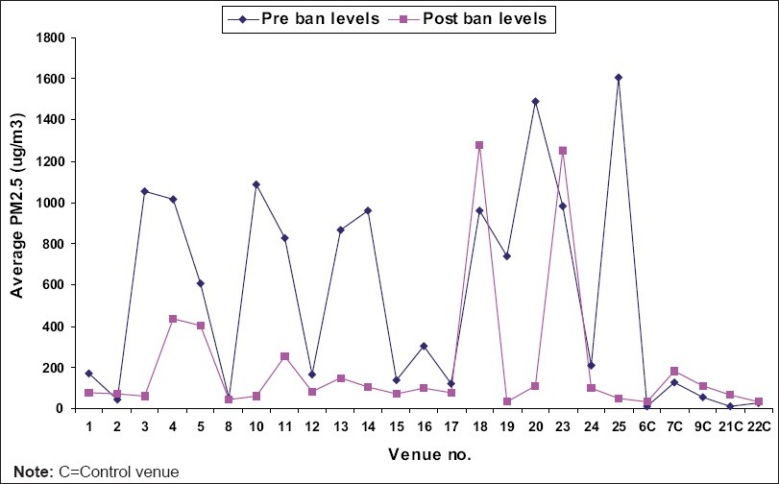
Comparison of pre and post-ban average levels of PM_2.5_

PM_2.5_ mean concentration in the two hookah parlors recorded post ban was 1267 μg/m^3^. This represents an almost 30% increase over the baseline concentration (973 μg/m^3^). Hookah parlors remained uniquely insulated from the ban’s effect. The PM_2.5_ levels recorded at all locations pre and post-comprehensive ban are graphically shown in Figure 4.

### Statistical analysis

The primary purpose was to assess the difference in the average PM_2.5_ in hospitality venues after implementation of the comprehensive public smoking ban. Differences between the average PM_2.5_ for each venue before and after implementation of the comprehensive public smoking ban were analyzed using the paired *T* test comparison. For average PM_2.5_ levels, the *P* value was calculated as 0.0019 whereas *P* value for the average smoker density was 0.0018. By conventional statistical criteria, this difference was very significant implying that smoking ban in hospitality venues had appreciably reduced the levels of PM_2.5_.

## Discussion

It was observed that the geographical location of venues did not affect the readings. This is due to the city’s more or less uniform population density and road traffic, regardless of vicinity to railway stations and sites with intense commercial activity.

‘No smoking’ signs were seen at all venues. However, cigarette smoking was observed in two hookah parlors (test venues 18 and 23) even after the smoking ban. It was apparent that cigarette smoking was not discouraged in the hookah enclosures. Venue 18 showed an exceptionally high smoker density. On average, up to four patrons shared every water-pipe. The ventilation was sub-optimal, with a 1 sq foot exhaust and two large conditioned vents. A visible smoke cloud hung in the lounge. This shift from cigarette smoking to hookah seemed to defeat the purpose of a ban on smoking.(13)

After the ban, smoking continues to be among the primary sources of particulate indoor air pollution but at a slightly lesser intensity. Adherence to the enacted legislation has been near total, with only pockets of local anomalies. Reports from countries like New Zealand, Romania, Lebanon, the United States and more(11) also suggest that smoke-free legislation led to air quality improvement in indoor public venues. These results were vindicated by significant declines in the number of cardio-pulmonary health complaints among hospitality employees(14,15) as also by the reduction in the hazardous breakdown products of tobacco in their saliva.(16)

Although legislation banning smoking had not been passed prior to these measurements in India, some restaurants had, out of tradition, prohibited patrons from smoking.(17) This one of its kind report on air particulates related to tobacco smoke before and after the ban has provided a ready reckoner for Public Health Welfare organizations and the medical fraternity equally. Whilst the overall environmental impact of anti-smoking legislation is positive, the onus is on the policy makers to execute it successfully and bring about improvements as required.
